# Kinetochore Architecture Employs Diverse Linker Strategies Across Evolution

**DOI:** 10.3389/fcell.2022.862637

**Published:** 2022-06-20

**Authors:** Shreyas Sridhar, Tatsuo Fukagawa

**Affiliations:** Laboratory of Chromosome Biology, Graduate School of Frontier Biosciences, Osaka University, Suita, Japan

**Keywords:** chromosome segregation, mitosis, kinetochore, centromere, evolution, CCAN, constitutive centromere associated network, mitotic spindle

## Abstract

The assembly of a functional kinetochore on centromeric chromatin is necessary to connect chromosomes to the mitotic spindle, ensuring accurate chromosome segregation. This connecting function of the kinetochore presents multiple internal and external structural challenges. A microtubule interacting outer kinetochore and centromeric chromatin interacting inner kinetochore effectively confront forces from the external spindle and centromere, respectively. While internally, special inner kinetochore proteins, defined as “linkers,” simultaneously interact with centromeric chromatin and the outer kinetochore to enable association with the mitotic spindle. With the ability to simultaneously interact with outer kinetochore components and centromeric chromatin, linker proteins such as centromere protein (CENP)-C or CENP-T in vertebrates and, additionally CENP-Q^Okp1^-U^Ame1^ in yeasts, also perform the function of force propagation within the kinetochore. Recent efforts have revealed an array of linker pathways strategies to effectively recruit the largely conserved outer kinetochore. In this review, we examine these linkages used to propagate force and recruit the outer kinetochore across evolution. Further, we look at their known regulatory pathways and implications on kinetochore structural diversity and plasticity.

## 1 Introduction

**FIGURE 1:**
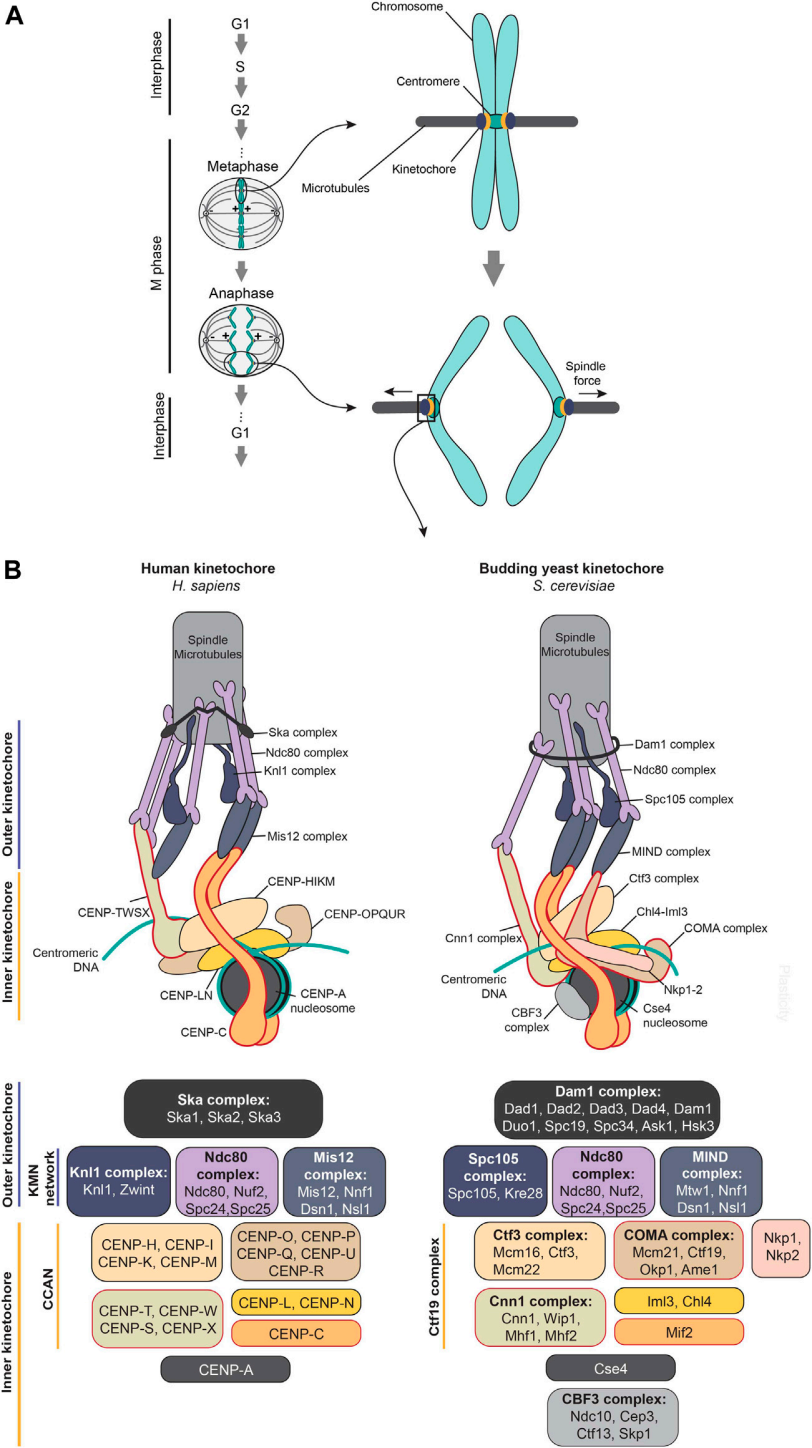
Schematic of kinetochore composition and architecture in human and budding yeast systems. **(A)** A functional kinetochore is assembled in M-phase on centromeric chromatin and facilitates interaction with spindle microtubules to ensure accurate chromosome segregation. **(B)** The kinetochore ensemble comprises of the inner and outer kinetochore networks. The constitutive centromere associated network (CCAN) at the inner kinetochore ensures the adequate recruitment of the outer kinetochore KMN network through specific linker proteins such as CENP-T and CENP-C in vertebrates and also CENP-Q^Okp1^-U^Ame1^ in budding yeast (highlighted in a bold maroon border). Although kinetochores across eukaryotes function to ensure accurate chromosome segregation, plasticity across its composition and architecture is observed which is more pronounced amongst inner kinetochore components. Homologous complexes between human and budding yeast kinetochore components have the same color codes. Kinetochore homologs have been mentioned in the corresponding positions.

The kinetochore is a macromolecular protein complex that forms on centromeric chromatin and couples forces from the mitotic spindle to facilitate accurate chromosome segregation (Figure 1A). Initial electron microscopy (EM) observations of the kinetochore architecture identified an inner and outer plate that was separated by a translucent layer (Luykx, 1965; Brinkley and Stubblefield, 1966). Spindle microtubules were observed to terminate on the outer plate. In recent years, we broadly distinguish the plates as protein networks of the inner, proximal to centromeric chromatin, and outer kinetochore, proximal to spindle microtubules (Figure 1B). What manifests itself as the translucent layer is yet not understood. Additionally, even after more than 50 years of studying the kinetochore a wholistic structural picture of this elegant structure has not been understood, while researchers in the field are just beginning to understand its plasticity. However, recent cryo-EM studies of the reconstituted inner kinetochore complex in budding yeast and humans are providing a strong platform for understanding kinetochore architecture and its evolutionary divergence (Hinshaw and Harrison, 2019; Yan et al., 2019; Pesenti et al., 2022; Yatskevich et al., 2022), Although the primary function of the kinetochore is to form load-bearing attachments, the kinetochore has also to control the feedback mechanism for the correction of inaccurate microtubule attachments through the recruitment of the components involved in the error correction mechanism and the spindle assembly checkpoint (SAC) (Foley and Kapoor, 2013; Joglekar and Kukreja, 2017; Lara-Gonzalez et al., 2021). Additionally, kinetochores are required to ensure their self-preservation across generations at the centromere through CENP-A replenishment (Black and Cleveland, 2011; McKinley and Cheeseman, 2016; Mellone and Fachinetti, 2021). To achieve these functional goals the kinetochore consists of more than 100 proteins, including around 30 core structural components (Figure 1B) (Cheeseman, 2014; Fukagawa and Earnshaw, 2014; Musacchio and Desai, 2017). This functionally conserved protein complex assembles on a variable centromeric platform that is not only some of the most rapidly evolving DNA sequences in the genome but is also diverse in terms of its organization, a contradiction termed the centromere paradox (Henikoff, 2001). Centromeric DNA varies, from the short ∼125 bp sequence-defined point centromeres of budding yeast to several Mb long repetitive regional centromeres in humans. Although chromosomes containing centromeres at a single locus, called monocentric chromosomes, were the first discovered, more recently systems ranging from plants to insects have been identified as consisting of kinetochore attachment sites across the length of a chromosome, defined as holocentric chromosomes (Boveri and Fischer, 1888; Clarke and Carbon, 1980; Buscaino et al., 2010; Fukagawa and Earnshaw, 2014; Guin et al., 2020).

In the last ∼30 years, great progress has been made towards the identification of kinetochore components, analysis of sub-complex functions, and their organization at the kinetochore across several model systems. Through these studies, the structural components of the kinetochore can be broadly classified into inner and outer layers. The centromere-specific histone H3 variant and hereditary factor CENP-A and the constitutive centromere associated network (CCAN) form the centromeric chromatin proximal inner layer (De Rop et al., 2012; Fukagawa and Earnshaw, 2014; McKinley and Cheeseman, 2016). While the outer kinetochore is comprised of the microtubule interaction facilitating KMN (Knl1, Mis12, and Ndc80 complexes) network (Figure 1B) (Musacchio and Desai, 2017).

**FIGURE 2:**
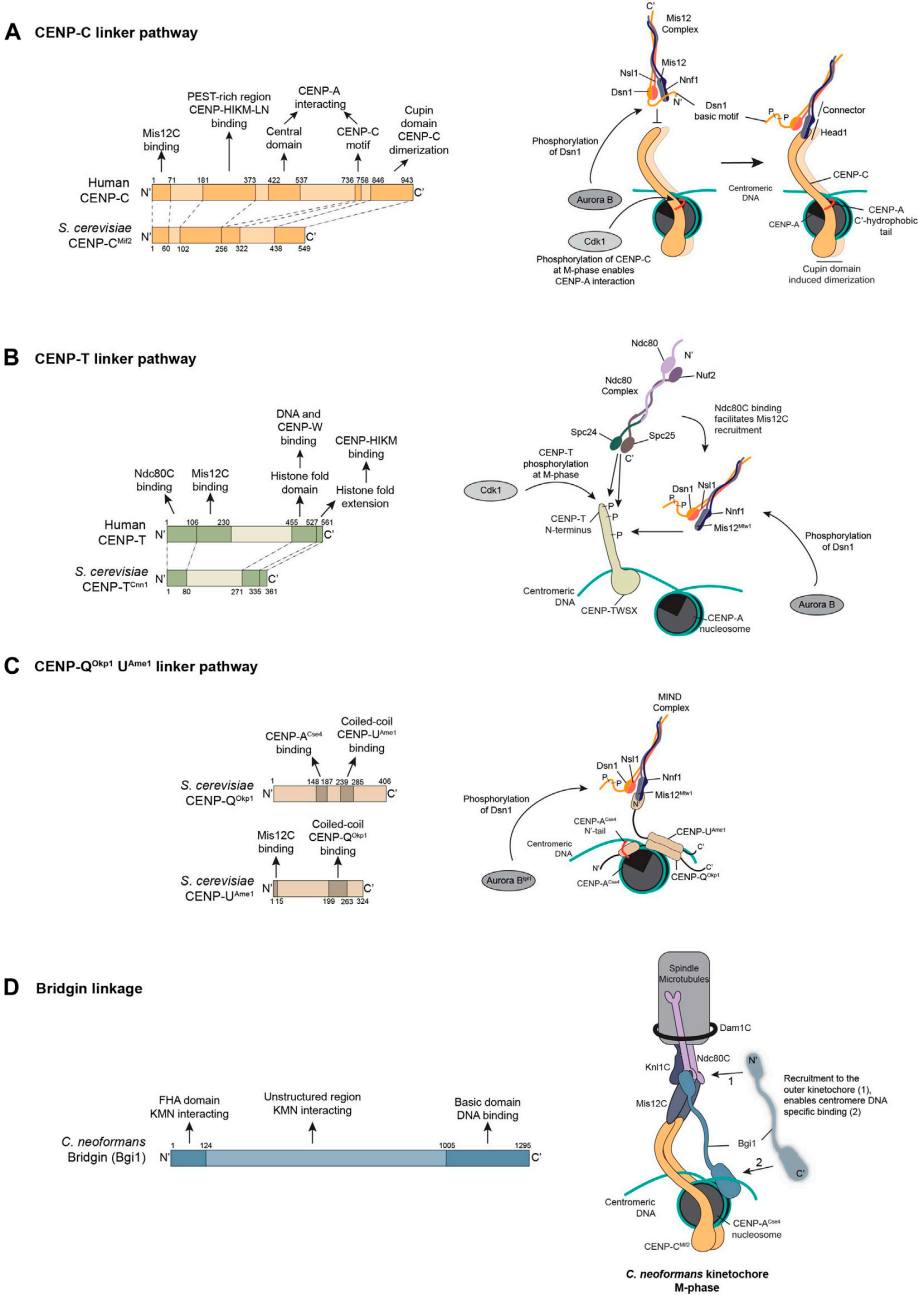
Linker pathways connect the outer kinetochore to centromeric chromatin. **(A)** The CENP-C linker pathway originates through the interactions of CENP-C with the C-terminal hydrophobic tail of CENP-A at the inner kinetochore. Subsequently, CENP-C through its N-terminal motif interacts with the Mis12-Nnf1 head of the Mis12C. This interaction is weakened/inhibited by the Dsn1 basic motif that binds to Mis12 and diminishes interaction with CENP-C in its unphosphorylated form. Aurora B-dependent phosphorylation alleviates this autoinhibition. **(B)** CENP-T complex interacts with centromeric linker DNA through a nucleosome-like structure formed by the histone-fold domains of CENP-T-W-S-X. At the N-terminus, in the human CENP-T, two Ndc80C recruitment sites exist which is under the control of Cdk1 phosphorylation. This Ndc80C binding subsequently facilitates the recruitment of the Mis12C. The phosphorylated form of Mis12C by Aurora B preferentially binds to CENP-T. **(C)** CENP-Q^Okp1^-U^Ame1^ has been described to serve as a linker pathway in budding yeast exclusively. While CENP-Q^Okp1^ interacts with the CENP-A^Cse4^ N-terminal tail, CENP-Q^Ame1^ has been described to interact with the Mis12^Mtw1^-Nnf1 head similar to CENP-C^Mif2^ ensuring the recruitment of the Mis12C^MIND^. **(D)** In *C. neoformans*, CENP-C^Mif2^ is the only conventional linker pathway described. Interestingly, a Ki-67-like protein named bridgin (Bgi1) was identified which is recruited to the outer kinetochore by the KMN network. This kinetochore-specific recruitment facilitates Bgi1 to subsequently interact with centromeric chromatin through its basic C-terminal motif. Thus generating a linkage between the outer kinetochore and centromeric chromatin.

The 16-member CCAN consists of CENP-C, CENP-L-N, CENP-H-I-K-M, CENP-T-W-S-X, and CENP-O-P-Q-U-R subcomplexes in vertebrates (Figure 1B) (Hara and Fukagawa, 2017). Although having distinct sub-complex functions, overall, the CCAN works to recuit and maintain centromeric CENP-A, mediate chromosome congression, and recruit the outer kinetochore components. Key CCAN components with the ability to simultaneously interact with centromeric chromatin and the outer kinetochore, such as CENP-T, CENP-C, or CENP-Q^Okp1^-U^Ame1^ in yeasts are defined as linkers in this review. Although being assisted by other kinetochore components in various capacities, it is only these known linker proteins that not only can recruit the outer kinetochore but also propagate spindle forces from them and transmit it to the underlying centromeric chromatin (Figures 1B, 2A–C). Thereby, these linker pathways form critical pillars to establish a functional kinetochore.

The outer kinetochore functions as the primary site for spindle microtubule-binding and is chiefly made up of the 10-member KMN network comprising the Knl1 complex (Knl1C), Mis12 complex (Mis12C), and Ndc80 complex (Ndc80C) (Lampert and Westermann, 2011; Musacchio and Desai, 2017). More recently, the structure of several of these sub-networks of proteins has been resolved and the kinetochore particle reconstituted, although several questions persist (Akiyoshi et al., 2010; Dimitrova et al., 2016; Petrovic et al., 2016; Weir et al., 2016; Hinshaw and Harrison, 2019; Yan et al., 2019; Pesenti et al., 2022; Yatskevich et al., 2022). Interestingly, the single kinetochore module present on the budding yeast point centromere seems to occur multivalently across larger regional centromeres where they are observed to form multiple kinetochore-microtubule attachments (Figure 1B). What started as a study in understanding a functionally conserved chromosome segregation machinery in defined model systems has grown today into an exploration towards understanding the diversity and rapid evolution of this system while retaining its functional conservation (Drinnenberg et al., 2016). With the improvement in genomic sequencing and bioinformatic tools, it is evident that the outer kinetochore components are rather well conserved across eukaryotic evolution (D’Archivio et al., 2017; Hooff et al., 2017; Plowman et al., 2019). By contrast, the platform that recruits it, the inner kinetochore, has undergone greater diversity, and components at the inner kinetochore have evolved a multitude of strategies to recruit the outer kinetochore to establish a functional kinetochore (Meraldi et al., 2006; Hooff et al., 2017). The last eukaryotic common ancestor (LECA) is suspected to contain a full compliment of the CCAN, including known linker components (Tromer et al., 2019). However, it is clear from recent comparative genomic studies that some CCAN components are often subsequently lost during evolution. Through this, the CENP-C linker pathway arises as the most conserved across evolution. The CENP-Q^Okp1^-U^Ame1^ linker pathway is exclusive to budding yeast. While components of the CENP-T pathway are recurrently lost across eukaryotic evolution (Hooff et al., 2017; Plowman et al., 2019). Being highly diverged, identification of homologs for these CCAN components is challenging and further analysis may identify additional homologs. However, biochemical analysis of Kinetoplastids kinetochores has revealed a whole new complement of kinetochore components validating the loss of all known kinetochore proteins (Akiyoshi and Gull, 2014; Ishii and Akiyoshi, 2022). Further predictions in Metamonads, Diplonemids and Euglenids suggest a similar loss event of most known kinetochore components (Butenko et al., 2020; Salas-Leiva et al., 2021; Tromer et al., 2021). Driven by recent exciting findings, in this review we explore the plasticity and mechanisms of how the inner kinetochore is set up during mitotic progression to recruit the essential outer kinetochore from available biochemical, and comparative genomic studies.

## 2 Outer Kinetochore and the KMN Network

The 10-member KMN network consisting of the Knl1C, Mis12C, and Ndc80C is predicted to form the most structurally conserved section of the kinetochore (Figure 1B). With Nuf2 and Ndc80, components of the Ndc80C, being the best conserved of them and only predicted to be lost in certain species of the group Euglenazoa and Metamonada, while the Mis12C is additionally lost in Apicomplexans (D’Archivio et al., 2017; Hooff et al., 2017; Plowman et al., 2019; Butenko et al., 2020; Brusini et al., 2021a; Salas-Leiva et al., 2021). The KMN network conservation is likely a consequence of its function in forming the primary interface with the near-ubiquitous segregation force generator, the spindle microtubules. The KMN network not only facilitates end-on attachments but also tracks depolymerizing microtubules, in turn transducing spindle forces to move chromosomes. Although functioning together to form accurate kinetochore-microtubule attachments, components of the KMN network: the Knl1C, comprising Knl1 and Zwint-1, the Mis12C, comprising Mis12, Dsn1, Nnf1, and Nsl1, and the Ndc80C, comprising of Spc24, Spc25, Nuf2, and Ndc80 (Figure 1B), have distinct functional roles. A number of extensive reviews have discussed the structure and function of the KMN network and thus we will only briefly describe the complexes (Lampert and Westermann, 2011; Cheeseman, 2014; Musacchio and Desai, 2017).

The 4-member Ndc80C, which is an ∼55 nm long-heterotetramer, forms the main microtubule contact site (Figure 1B) (Cheeseman et al., 2006; DeLuca et al., 2006). The complex is comprised of two dimers Nuf2-Ndc80, and Spc24-Spc25 that are held together by the overlapping α-helical coiled-coil domains in the C-termini of Nuf2-Ndc80 and N-termini of Spc24-Spc25 (Wei et al., 2007; Cheeseman and Desai, 2008; Ciferri et al., 2008). The Nuf2 and Ndc80 subunits each contain calponin-homology (CH) domains at their N-termini that are tightly packed in the Ndc80C structure to mediate microtubule binding (Ciferri et al., 2008; Valverde et al., 2016; Wei et al., 2007). Additionally, the highly disordered basic N-terminal tail of Ndc80 has been implicated in microtubule interactions (Ciferri et al., 2008; Guimaraes et al., 2008; Miller et al., 2008). Kinetochore targeting of the Ndc80C is mediated by Spc24-Spc25 through interactions with either CENP-T or Dsn1-Nsl1 subunits of the Mis12C (Figure 2B) (Petrovic et al., 2010; Gascoigne et al., 2011; Bock et al., 2012; Schleiffer et al., 2012; Hori et al., 2013; Malvezzi et al., 2013; Nishino et al., 2013; Petrovic et al., 2016).

The ∼20 nm long rod-shaped Mis12C, also known as the MIND complex in *S. cerevisiae*, forms the scaffold enabling the nucleation of the KMN network *via* harboring binding sites for both Ndc80C and Knl1C (Figure 1B) (Maskell et al., 2010; Petrovic et al., 2010). The Mis12C heterotetramer is formed by dimers of Dsn1-Nsl1, and Mis12-Nnf1. The subunits are structural paralogs having high helical content (Figure 2A). Linear motifs close to the C-termini of Nsl1 and Dsn1 provide binding sites to the RWD domains present in the Ndc80C subunits of Spc24-Spc25. The Nsl1 C-terminal tail in addition to an extended interface generated by the C-terminal four-helix bundle in the stalk of the Mis12C enables interaction with the Knl1C (Petrovic et al., 2010; Dimitrova et al., 2016; Petrovic et al., 2016).

Knl1C is a heterodimer of Knl1 (Spc105 in fungi) and Zwint-1 or its homolog Kre28/Sos7 in fungi. Knl1 is a largely disordered protein with a coiled-coil region followed by the tandem RWD domains at its C-terminus (Figure 1B). The RWD domain facilitating protein-protein interactions is a recurring module at the kinetochore, with up to eight kinetochore proteins harbouring it (Schmitzberger and Harrison, 2012; Tromer et al., 2019). The coiled-coil domain on Knl1 plays host to the interaction with Zwint-1, while the RWD domain mediates interactions with the Mis12C (Petrovic et al., 2010; Petrovic et al., 2014; Petrovic et al., 2016). The largely unstructured N-terminal region of Knl1 comprises an array of protein docking sites, recruiting proteins such as PP1 and Bub1-Bub3 critical in regulating kinetochore dynamics, activating SAC, and in error correction (Kiyomitsu et al., 2007; Cheeseman and Desai, 2008). Towards SAC activation, Bub1-Bub3 is recruited through Met-Glu-Leu-Thr (MELT) repeats following the phosphorylation of the conserved Thr residue by Mps1 kinase (Joglekar, 2016). Additionally, residues in the extreme N-terminus are also involved in microtubule-binding and bundling which is required for checkpoint silencing (Espeut et al., 2012; Bajaj et al., 2018).

Outside of the KMN network, the outer kinetochore also consists of accessory factors that aid in tracking microtubules during the M-phase (Cheeseman, 2014). The functionally analogous 10-member Dam1 complex (Dam1C) or the 3-member Ska complex (SkaC) performs this function and is found to be widespread, but exceptionally inverse in their conservation across eukaryotic evolution (Figure 1B) (Hooff et al., 2017). The Dam1C is abundantly conserved across fungi, while the SkaC is observed in other systems. It is also suggested that components of the Dam1 complex are analogs of the Ska complex, likely existing in the LECA (van Hooff et al., 2017). Being out of the scope of this review further insights into these complexes can be found in other publications (Jeyaprakash et al., 2012; Cheeseman, 2014; Abad et al., 2016; van Hooff et al., 2017; Jenni and Harrison, 2018).

Although being largely conserved across eukaryotic evolution and playing a critical role in microtubule binding and SAC recruitment, a single ubiquitously conserved mechanism to ensure KMN network recruitment at the kinetochore is not observed rather a diversity of linkages has been reported across eukaryotes (discussed below), with systems regularly hosting multiple pathways.

## 3 Inner Kinetochore and the CCAN

The inner kinetochore comprises the centromere-specific histone H3 variant CENP-A and the 16-member CCAN in vertebrates (Figure 1B) (Hara and Fukagawa, 2017). In the budding yeast system, the CCAN is referred to as the Ctf19 complex (Ctf19C), wherein homologs for CENP-M and CENP-R are absent (Figure 1B). The Ctf19C in addition contains Nkp1-Nkp2 proteins, which share ancestry with the Mis12C^MIND^ (Figure 1B) (Biggins, 2013; Tromer et al., 2019). While CENP-A marks an active centromere in most species, recent studies have identified species in which CENP-A is lost and does not exist on a centromeric locus that forms a functional kinetochore. Of the recently identified CENP-A-deficient systems, *Bombyx mori* and *Mucor circinelloides* have retained CCAN components (Navarro-Mendoza et al., 2019; Cortes-Silva et al., 2020), while kinetoplastids have in addition lost all known kinetochore components (Akiyoshi and Gull, 2014; Ishii and Akiyoshi, 2022). CENP-A, when present, forms a nucleosome structure in which the canonical histone H3 is replaced with CENP-A. Much debate has surrounded as to what makes the CENP-A chromatin “special,” summarized in other studies (McKinley and Cheeseman, 2016; Ali-Ahmad and Sekulić, 2020; Mitra et al., 2020a). CCAN components CENP-C and CENP-N in vertebrates directly bind to the CENP-A nucleosome to assemble the whole CCAN structure in the centromeric chromatin (Carroll et al., 2009; Kato et al., 2013; Watanabe et al., 2019; Ariyoshi et al., 2021), and CENP-U^Ame1^-Q^Okp1^ in budding yeast also directly binds to the CENP-A^Cse4^ nucleosome (Anedchenko et al., 2019; Fischböck-Halwachs et al., 2019). This recognition of centromeric chromatin by CCAN factors facilitates the formation of the kinetochore. How the kinetochore assembles on CENP-A deficient centromeres is unknown and a critical question in this field.

Cryo-EM structures of the human (Pesenti et al., 2022; Yatskevich et al., 2022) and yeast (Hinshaw and Harrison, 2019; Yan et al., 2019) CCAN complexes have greatly aided in our understanding of its structure-function. The CCAN is observed as a defined complex where its subunits interdigitate rather than forming a network of binary interactions. Further, the Y-shaped opening of the budding yeast CCAN^Ctf19C^ cradles the CENP-A^Cse4^ nucleosomes on either side in a ratio of 2:1. A conserved feature across systems is the strong binding to linker DNA by the CENP-L-N channel. Reminiscent of canonical nucleosomes the CENP-T-W-S-X and CENP-H-I-K modules partially wrap linker DNA. Thus, the CCAN through its tight entrapment of linker DNA provides insights into how the strong push-pull forces of the mitotic spindle are handled by the inner kinetochore. While the formation of the CCAN on centromeric chromatin is critical, it is insufficient for a full kinetochore activity. This requires the recruitment of the microtubule interacting outer kinetochore by linker members of the CCAN.

## 4 Linkages Connecting the Outer Kinetochore to Centromeric Chromatin

As linkers, CENP-C, CENP-T, or CENP-Q^Okp1^-U^Ame1^ function to recruit the outer kinetochore either as a complete KMN network unit, through the interaction of the Mis12C with CENP-C/-T/-Q^Okp1^-U^Ame1^, or only the Ndc80C through the direct interaction with the N-terminal region of CENP-T (Figures 2A–C). In addition, these linker pathways also function to effectively transmit forces from the spindle-bound outer kinetochore to chromosomes, manifesting as “stretch” in the disordered regions in CENP-T and CENP-C (Hara et al., 2018; Suzuki et al., 2011; Suzuki et al., 2014; Uchida et al., 2021; Ye et al., 2016). At the inner kinetochore, the linker pathways of CENP-C and CENP-Q^Okp1^-U^Ame1^ contact centromeric chromatin through CENP-A^Cse4^ (Figures 2A,C) (Anedchenko et al., 2019; Ariyoshi et al., 2021; Fischböck-Halwachs et al., 2019; Kato et al., 2013). While, the CENP-T complex has been described to bind centromeric DNA and induce supercoiling in it (Figure 2B) (Hori et al., 2008a; Nishino et al., 2012; Takeuchi et al., 2014; Pesenti et al., 2022; Yatskevich et al., 2022). The multifaceted roles of linker proteins at the kinetochores make them key candidates for regulatory action (Hara and Fukagawa, 2019). Thus, linker proteins function as a critical junction between centromeric chromatin and the outer kinetochore. Here we summarize the detailed understanding of each of the defined linkages across model systems (Figures 2A–D).

### 4.1 The CENP-C Pathway

CENP-C, along with CENP-A and CENP-B, was identified as an antigen detected by the sera from patients diagnosed with the autoimmune syndrome CREST (Calcinosis, Reynaud’s syndrome, Esophageal dysmotility, Sclerodactyly, Telangiectasia) (Moroi et al., 1980). The following works characterized CENP-C as a centromere protein and later an inner kinetochore component required for cell cycle progression and in the maintenance of the kinetochore’s trilaminate structure (Earnshaw and Rothfield, 1985; Saitoh et al., 1992; Tomkiel et al., 1994; Fukagawa and Brown, 1997; Kalitsis et al., 1998). Thus, cementing CENP-C as the first described component of the kinetochore.

CENP-C has multiple conserved domains which enable interaction with the outer kinetochore, multiple CCAN components, and the CENP-A nucleosome (Figure 2A). These include the N-terminal domain for Mis12C binding (Gascoigne et al., 2011; Hori et al., 2013; Petrovic et al., 2016; Przewloka et al., 2011; Screpanti et al., 2011), a middle conserved region that binds CENP-H-I-K-M and -L-N (Klare et al., 2015; McKinley et al., 2015; Nagpal et al., 2015), two CENP-A binding motifs in humans (Carroll et al., 2010; Kato et al., 2013; Guo et al., 2017), with one each in chicken and budding yeast (Cohen et al., 2008; Watanabe et al., 2019; Ariyoshi et al., 2021), and an extreme C-terminal cupin domain involved in CENP-C dimerization (Figure 2A) (Cohen et al., 2008; Trazzi et al., 2009). Owing to its extensive interaction at the kinetochore, CENP-C has been proposed to form a central hub for kinetochore assembly in human cells (Klare et al., 2015; Weir et al., 2016).

#### 4.1.1 CENP-C Dynamics at the Inner Kinetochore

CENP-C was first described to have DNA binding capability (Yang et al., 1996) and later shown to interact specifically with the CENP-A nucleosome by recognizing its C-terminal hydrophobic tail, distinguishing it from canonical histone H3 (Figure 2A) (Westermann et al., 2003; Xiao et al., 2017; Kato et al., 2013). A recent work using *in vitro* reconstituted human full-length CENP-C from the Musacchio lab describes it to dimerize and bind simultaneously to two CENP-A nucleosomes in humans (Walstein et al., 2021). Interestingly this interaction of CENP-C with CENP-A was shown to be transient, occurring exclusively in M-phase, controlled by CDK1 phosphorylation in both chicken and human cells (Figure 2A) (Nagpal et al., 2015; Watanabe et al., 2019; Ariyoshi et al., 2021). Strikingly, chicken CENP-C contains a single CENP-A binding site, the CENP-C motif, which happens to be dispensable for cell viability. Although this motif is dispensable in human cells too, simultaneous disruption of a second CENP-A interacting motif, the central domain, is not (Watanabe et al., 2019). Unlike in the vertebrate systems, the sole CENP-A^Cse4^ contact site, the CENP-C motif, is essential in budding yeast (Cohen et al., 2008; Hornung et al., 2014). This difference in CENP-C requirement at the inner kinetochore across systems further manifests itself in the localization hierarchy of CCAN components. A greater dependence of the CENP-H-I-K-M and CENP-T-W-S-X complexes on CENP-C is observed in budding yeast, human and *Xenopus* systems over chicken cells, but is not correlated with its function as a linker protein (see subsequent sections) (Westermann et al., 2003; Hori et al., 2008a; Krizaic et al., 2015; McKinley et al., 2015; Weir et al., 2016).

Although not constitutively bound to the CENP-A nucleosome, CENP-C is suggested to be recruited to the kinetochore in G1 in coordination with CENP-A recruitment in *Xenopus* (Krizaic et al., 2015). Outside M-phase, CENP-C interacts with CENP-H-I-K-M and CENP-L-N through its middle conserved region. This interaction retains CENP-C stably at the kinetochore in humans cells, while is described to be dynamic in chicken cells (Hemmerich et al., 2008; Nagpal et al., 2015; Watanabe et al., 2022). Additionally, CENP-B has been shown to preserve CENP-C anchoring at the centromere in the absence of CENP-A contacts in human cells (Hoffmann et al., 2016).

#### 4.1.2 CENP-C Interaction With the Outer Kinetochore

Once anchored at the centromere, CENP-C can function to recruit and transmit KMN network-generated force (Ye et al., 2016). CENP-C through its ∼45-residue N-terminal motif contacts the Mis12C (Przewloka et al., 2011; Screpanti et al., 2011; Hornung et al., 2014; Liu et al., 2016; Richter et al., 2016). In the budding yeast, *Kluyveromyces lactis*, and in the fruit fly *Drosophila melanogaster*, CENP-C contacts the N-terminal head domain of Mis12 and Nnf1. Whereas in the human homolog a composite site comprising the head domain and helical connector of Dsn1-Nsl1 was shown to be required. Through these interactions, each CENP-C molecule has the potential to recruit a single KMN unit at the kinetochore (Figure 2A) (Dimitrova et al., 2016; Petrovic et al., 2016; Richter et al., 2016).

This interaction between CENP-C and the Mis12C is regulated by Aurora B kinase-dependent phosphorylation. In an intra-Mis12C manner the N-terminal basic motif residing in a disordered region of Dsn1 masks the CENP-C interaction site on the Mis12C in its unphosphorylated state. This limits the interaction between CENP-C and the Mis12C. Upon phosphorylation by Aurora B kinase, the inhibitory motif moves away from the interaction site, allowing for ∼150-fold increased binding affinity of the Mis12C to CENP-C (Figure 2A) (Welburn et al., 2010; Kim and Yu, 2015; Dimitrova et al., 2016; Petrovic et al., 2016). As a part of this regulatory cycle, the phosphorylation is suggested to be countered by PP1, more so during M-phase induced stretch when the Mis12C is further away from the influence of centromere-localized Aurora B. This may further be magnified in anaphase when Aurora B relocalizes to the midzone (Hara et al., 2018; Lang et al., 2018). Interestingly, the *D. melanogaster* Dsn1 homolog lacks the basic inhibitory motif in its N-terminal region, thus eliminating this autoinhibition at its kinetochore.

Consistent with the ability of the CENP-C N-terminus to recruit a full complement of the KMN network, artificial tethering experiments of this region to a non-centromeric locus by the LacO-LacI system in chicken DT40 cells and human cell lines were able to ensure the normal segregation of the tethered chromosome (Gascoigne et al., 2011; Hori et al., 2013). Importantly no other CCAN components were localized to the tethered site. This suggests that at the ectopic loci the CENP-C N-terminus was necessary and sufficient for recruiting the KMN network and in forming functional kinetochore-microtubule attachments through the extensive multicopy tethering system (Gascoigne et al., 2011; Hori et al., 2013). Thus, to prevent the unregulated recruitment of a functional outer kinetochore unit on rogue CENP-C at a non-centromeric locus, the Westermann lab recently reported that CENP-C^Mif2^ autoinhibition prevents interaction with the Mis12C^MIND^ in the absence of CENP-A^Cse4^ binding in budding yeast cells (Killinger et al., 2020).

### 4.2 The CENP-T Pathway

CENP-T was identified as a component of the CENP-A containing centromere complex (Obuse et al., 2004; Foltz et al., 2006; Izuta et al., 2006). Subsequent studies from our lab established it as a key component of the inner kinetochore CCAN network required for the recruitment of KMN network components (Figure 2B) (Hori et al., 2008a; Nishino et al., 2012, 2013; Hara et al., 2018; Takenoshita et al., 2022). Initially, the budding yeast kinetochore was thought to lack CENP-T and be divergent from vertebrate kinetochores. However, robust computations analysis identified the CENP-T homolog as the budding yeast protein Cnn1 (Schleiffer et al., 2012). Interestingly, Cnn1 was identified before the discovery of the vertebrate CENP-T as “copurified with Nnf1” (De Wulf et al., 2003) and was suggested to be a point centromere-specific protein (Meraldi et al., 2006). The difficulty in recognizing CENP-T homologs, similar to other CCAN components, highlights their sequence divergence across evolution (Hooff et al., 2017).

CENP-T is the dominant protein of its namesake complex. A functional CENP-T complex has been shown to comprise the CENP-T-W (Hori et al., 2008a) and CENP-S-X (Amano et al., 2009) dimers. Each subunit contains a histone fold (Nishino et al., 2012). Phylogenetic analysis has suggested that CENP-T-W-S-X may have arisen after two duplication events of CENP-S-T and CENP-X-W, which is interconnected with the origin of the eukaryotic transcription and DNA repair machinery (Tromer et al., 2019). This is owing to the role of CENP-S-X in the Fanconi Anaemia pathway (Singh et al., 2010; Yan et al., 2010).

#### 4.2.1 CENP-T Dynamics at the Inner Kinetochore

The CENP-T-W and CENP-S-X dimers can form a heterotetrameric nucleosome-like structure that can bind DNA and induce positive super-coiling, unlike conventional nucleosomes that generate negative supercoils at budding yeast mini-chromosomes (Furuyama and Henikoff, 2009; Nishino et al., 2012; Takeuchi et al., 2014; Pesenti et al., 2022; Yatskevich et al., 2022). Loss of CENP-S-X has shown not to strongly affect CENP-T-W levels at the kinetochore although CENP-S is sufficient to recruit CENP-T to an ectopic locus (Amano et al., 2009; Nishino et al., 2012).

Although being constitutively localized at centromeric chromatin, CENP-T has been shown to be rapidly turned over and not stability inherited at the human kinetochore (Prendergast et al., 2011). In budding yeast, CENP-T^Cnn1^ levels rapidly increase post anaphase onset which is regulated by multiple mitotic kinases (Bock et al., 2012). Loading of CENP-T-W at the human kinetochore has been suggested to take place in the S and G2 phase of the cell cycle, independent of DNA replication and CENP-A presence but requiring the H2A/B chaperone, FACT (Hoffmann et al., 2016; Prendergast et al., 2011, Prendergast et al., 2016).

Although the precise mechanism of CENP-T regulation and deposition at the kinetochore is not understood, studies have shown that the interaction of CENP-T-W with the CENP-H-I-K complex is critical for its stable retention. A major binding interface is formed between the conserved CENP-T C-terminal histone-fold extension α-helix and a CENP-K α-helix (Hori et al., 2008a; Nishino et al., 2012; Basilico et al., 2014; McKinley et al., 2015; Pekgöz Altunkaya et al., 2016; Hinshaw and Harrison, 2020; Zhang et al., 2020; Pesenti et al., 2022; Yatskevich et al., 2022). Although CENP-T interaction with CENP-H-I-K is essential, it is insufficient for CENP-T complex kinetochore localization, as point mutations that affect DNA binding in CENP-T-W also completely abolish CENP-T localization (Nishino et al., 2012; McKinley et al., 2015). Additionally, CENP-A tails have been shown to affect CENP-T kinetochore levels in fission yeast and in human cells during centromere establishment (Folco et al., 2015; Logsdon et al., 2015). Thus, addressing how the synergy between CCAN contacts and DNA binding helps maintain and recruit CENP-T at the kinetochore requires further investigation and would aid greatly in a holistic view of the inner kinetochore assembly. In addition to CCAN factors, recent studies have also implicated the SUMO protease SENP6 in maintaining CCAN protein levels including CENP-T and CENP-C at the kinetochore, consequently affecting CENP-A maintenance (Mukhopadhyay et al., 2010; Liebelt et al., 2019; Wagner et al., 2019; Mitra et al., 2020b).

The CENP-T complex being the only other histone-fold containing complex identified at the kinetochore, after CENP-A, raises the question as to how it is positioned within the kinetochore structure? Insights from *in vitro* studies using chicken proteins suggest that the CENP-T complex binds preferentially to an ∼100 bp linker DNA over nucleosome-bound DNA (Figure 2B) (Takeuchi et al., 2014). Indeed, the recent structure of the human CCAN described CENP-T-W-S-X together with CENP-H-I-K^Head^ to partially wrap around linker DNA reminiscent of canonical histones (Yatskevich et al., 2022), as previously shown with DNA and the recombinant CENP-T-W-S-X complex (Nishino et al., 2012; Takeuchi et al., 2014). This is consistent with ChIP analysis, positioning CENP-T between two CENP-A nucleosomes at the regional centromeres of humans and fission yeast (Thakur et al., 2015; Thakur and Henikoff, 2016). On the other hand, in the point centromere containing budding yeast, CENP-T^Cnn1^ is suggested to bind the core centromere particle, a region overlapping with CENP-A^Cse4^, and not form a separate nucleosome-like particle (Pekgöz Altunkaya et al., 2016; Hinshaw and Harrison, 2020; Zhang et al., 2020). Thus, the positioning of CENP-T at the kinetochore may vary to accommodate the constraints of the system differing from point to regional centromeres which might offer an explanation towards its variable interdependencies (Hori et al., 2008a; Pekgöz Altunkaya et al., 2016; Walstein et al., 2021).

#### 4.2.2 CENP-T Interaction With the Outer Kinetochore

CENP-T possesses a long unstructured N-terminal region, unlike the other small histone-fold containing proteins, CENP-W, -S, and -X of the CENP-T complex. The interaction of the CENP-T complex with the outer kinetochore occurs through multiple domains contained in this CENP-T N-terminal region (Figure 2B) (Bock et al., 2012; Schleiffer et al., 2012; Rago et al., 2015; Hara et al., 2018). At the extreme amino-terminal end two Ndc80C binding motifs in humans, and one each in chicken cells or budding yeast are present. Following this is the Mis12C binding site that ensures a complete KMN network ensemble on CENP-T (Emanuele et al., 2008; Nishino et al., 2013; Rago et al., 2015; Huisin’T Veld et al., 2016; Hara et al., 2018; Takenoshita et al., 2022). Both Ndc80C and Mis12C interactions with the CENP-T N terminus are regulated by multiple phosphorylation events (Figure 2B) (see below in detail). Interestingly, no Mis12C^MIND^ binding on the budding yeast CENP-T^Cnn1^ has been reported.

CENP-W is an integral interacting partner of CENP-T, and its absence has been shown to severely affect CENP-T at the kinetochore, inturn affecting outer kinetochore levels (Hori et al., 2008a; Bock et al., 2012; Nishino et al., 2012, Nishino et al., 2013; Schleiffer et al., 2012). Yet interestingly, in the *B. mori* system, biochemical searchers did not detect any homolog of CENP-W (Cortes-Silva et al., 2020). While in the *Xenopus* egg extract system, CENP-T and CENP-W might have variable temporal dynamics during *de novo* kinetochore formation (Krizaic et al., 2015).

On the other hand, CENP-S-X loss has been shown not to alter CENP-T levels at the kinetochore, yet they have been described to influence the localization of the outer kinetochore (Amano et al., 2009; Nishino et al., 2012). If this is through the CENP-T recruited KMN network is to be explored. While, in the moss system of *Physcomitrella patens*, the conditional knockdown of CENP-S-X results in segregation defects that phenocopies other outer kinetochore components although not exhibiting distinct kinetochore localization (Kozgunova et al., 2019). Thus, further analysis following these insights may lead to a better functional understanding of the CENP-T complex components.

Recruitment of the outer kinetochore by CENP-T in vertebrates occurs exclusively in the M-phase, with CDK1 phosphorylation-dependent binding of the Ndc80C. This binding of the Ndc80C onto CENP-T is necessary for subsequent recruitment of the Mis12C, which is also under the influence of CDK1 phosphorylation of CENP-T (Figure 2B) (Kettenbach et al., 2011; Nishino et al., 2013; Rago et al., 2015; Suzuki et al., 2015; Huisin’T Veld et al., 2016). In addition, Dsn1 of the Mis12C is also necessary to be phosphorylated by Aurora B kinase for stable binding to CENP-T (Figure 2B) (Walstein et al., 2021). In budding yeast, the Ndc80C recruitment may not be regulated by phosphorylation, but indirectly controlled through the phosphorylation-dependent increase of CENP-T^Cnn1^ itself at anaphase onset (Bock et al., 2012; Malvezzi et al., 2013). In an interesting twist, CENP-T^Cnn1^ has also been shown to be able to localize to the kinetochore *via* its N-terminal Spc24/25 interacting sequence (Thapa et al., 2015). Considering CENP-T^Cnn1^ and the Mis12C compete for the same Spc24/25 binding site. Which pool of the Ndc80C, the N-terminal CENP-T^Cnn1^ is recruited by is not known. Convergently, the Ndc80C binding site on CENP-T resembles that on Dsn1 (Malvezzi et al., 2013; Dimitrova et al., 2016; Hara et al., 2018). Yet, strikingly, the CDK1 phosphorylation which is required for Ndc80C recruitment on CENP-T reduces the affinity of the Ndc80C for Dsn1 (Rago et al., 2015). The intention of this regulation is not yet understood.

Previous studies propose that the Mis12C binds to CENP-T using an overlapping region that is also involved in CENP-C binding. Thus forcing CENP-T and CENP-C to compete for Mis12C binding (Huisin’T Veld et al., 2016). More recent work builds on these findings, suggesting that in addition, the Aurora B kinase-dependent phosphorylation that limits interactions between Mis12C and CENP-C might also be involved in the Mis12C-CENP-T interaction (Walstein et al., 2021). Overall, each CENP-T molecule, dependent on the system, is capable of recruiting 2 or 3 Ndc80C. One or two Ndc80C are recruited directly and an additional Ndc80C is recruited *via* the Mis12C binding onto CENP-T (Figure 2B) (Rago et al., 2015; Huisin’T Veld et al., 2016; Hara et al., 2018; Takenioshita et al., 2022).

To test the sufficiency of the CENP-T N-terminus in recruiting a functional outer kinetochore, tethering of this region at an ectopic site by the LacO-LacI system was performed. This tethering was found to be sufficient to segregate the chromosome harboring the tethered site in vertebrates systems, as well as in the context of an autonomous-replicating sequence (ARS) containing plasmid in budding yeast (Gascoigne et al., 2011; Schleiffer et al., 2012; Hori et al., 2013). Thus, like the CENP-C pathway, CENP-T *via* its N-terminus can recruit a functional unit of the outer kinetochore without the presence of any other CCAN at an ectopic locus, to ensure chromosome segregation.

### 4.3 The CENP-Q^Okp1^-U^Ame1^ Pathway in Budding Yeasts

*S. cerevisiae* harbors an almost complete complement of the CCAN^Ctf19C^ (Figure 1B). Yet, CENP-C^Mif2^, CENP-Q^Okp1^, and CENP-U^Ame1^ are the only CCAN^Ctf19C^ components that are essential for viability (Biggins, 2013)*.* CENP-Q^Okp1^ and CENP-U^Ame1^ form a dimeric subcomplex which along with CENP-O^Mcm21^ and CENP-P^Ctf19^ constitute the COMA complex (Hyland et al., 1999; Ortiz et al., 1999; Poddar et al., 1999; Cheeseman et al., 2002). CENP-Q^Okp1^ and CENP-U^Ame1^ contain a coiled-coil region in the C-terminal half and harbor no other distinct structural domains (Figure 2C) (Hornung et al., 2014). The Nkp1-Nkp2 heterodimer has been described to facilitate stabilization of the COMA complex through interactions with CENP-Q^Okp1^-U^Ame1^. The absence of Nkp1-Nkp2 in vertebrates may be substituted by its functional analog CENP-R (Schmitzberger et al., 2017; Hinshaw and Harrison, 2019; Yan et al., 2019).

Microscopic observation shows that CENP-Q^Okp1^ and CENP-U^Ame1^ are more abundant at the kinetochore than CENP-P^Ctf19^, suggesting a variable functional unit of the COMA complex (Dhatchinamoorthy et al., 2017). Although CENP-O^Mcm21^ and CENP-P^Ctf19^ are non-essential for viability, they are required for accurate chromosome segregation (Hyland et al., 1999). CENP-Q^Okp1^ and CENP-U^Ame1^ lie upstream to all but CENP-C^Mif2^ at the CCAN^Ctf19C^ assembly hierarchy. Their levels increase in anaphase and are amongst the most abundant CCAN^Ctf19C^ proteins at the kinetochore (Dhatchinamoorthy et al., 2017). While CENP-Q^Okp1^-UA^me1^ is a linker protein in *S. cerevisiae*, vertebrate CENP-Q-U bind neither to outer kinetochore components nor to the CENP-A nuclesome, and the CENP-O-P-Q-U-R complex is not required for cell viability in chicken DT40 cells (Okada et al., 2006; Hori et al., 2008b; Kagawa et al., 2014), suggesting that vertebrate CENP-Q-U does not function as a linker pathway.

#### 4.3.1 CENP-Q^Okp1^-U^Ame1^ Dynamics at the Inner Kinetochore

Unlike simultaneous interactions of CENP-T or CENP-C with centromeric chromatin and the outer kinetochore, a division of labor amongst the essential CENP-Q^Okp1^ and CENP-U^Ame1^ is observed. CENP-Q^Okp1^ interacts with centromeric CENP-A^Cse4^-contaning nucleosome while CENP-U^Ame1^ interacts with the Mis12C^MIND^ (Figure 2C) (Hornung et al., 2014; Anedchenko et al., 2019; Fischböck-Halwachs et al., 2019). CENP-Q^Okp1^-U^Ame1^ was initially described to interact with DNA which was not specific to the point centromeric sequences of budding yeast, suggesting other mechanisms to enable its centromere targeting (Hornung et al., 2014). Two recent studies (Anedchenko et al., 2019; Fischböck-Halwachs et al., 2019) describe in detail how CENP-Q^Okp1^-U^Ame1^ achieves this (Figure 2C). Fischböck-Halwachs et al. (2019) show that CENP-Q^Okp1^ of the heterodimer was sufficient to form direct binding selectively to the CENP-A^Cse4^ N-terminus. A 45-amino acid domain called the core domain of CENP-Q^Okp1^ (residues 127–184) was critical for this interaction. In addition, Anedchenko et al. (2019) found cross-links between the centromeric nucleosome and CENP-U^Ame1^ in the *in vitro* reconstituted complex. They also show that methylation on R37 and acetylation on K49 of CENP-A^Cse4^ inhibit interaction with CENP-Q^Okp1^-U^Ame1^, suggesting a reader-like function for the heterodimer.

#### 4.3.2 CENP-Q^Okp1^-U^Ame1^ Interaction With the Outer Kinetochore

Immunoprecipitation (IP) followed by mass-spectrometry (MS) analysis of CENP-U^Ame1^ revealed Mis12^Mtw1^ as the most abundantly interacting KMN network component in *S. cerevisiae*. Follow-up studies using *in vitro* reconstitution described a short conserved motif containing 15 residues at the extreme N-terminal of CENP-U^Ame1^ that was necessary and sufficient for interaction with the Mis12-Nnf1 globular head domain. The binding stoichiometry of CENP-U^Ame1^ to the Mis12C^MIND^ is 1:1. This interaction with the Mis12C^MIND^ was further aided by the co-operative binding of CENP-C^Mif2^ bringing the stoichiometry to 2:2:2 for CENP-C^Mif2^:CENP-Q^Okp^-^1^U^Ame1^:Mis12C^MIND^ at the kinetochore, after accounting for CENP-C^Mif2^ dimerization (Hornung et al., 2014). Subsequent structural analysis of *K. lactis* proteins suggested that CENP-C^Mif2^ and CENP-U^Ame1^ bind noncompetitively to the globular head domain of Mis12^Mtw1^-Nnf1 at distinct interfaces *in vitro* (Dimitrova et al., 2016). However, in disagreement, a recent study from the Westermann lab (Killinger et al., 2020), shows using *in vitro* and *in vivo* assays that CENP-U^Ame1^ and CENP-C^Mif2^ compete with each other and do not bind to the same Mis12C^MIND^ (Figures 2A,C). They further present evidence that instead of occupying spatially distinct sites on the Mis12C head I, they occupy largely overlapping or identical binding sites where mutations in the CENP-C^Mif2^ binding interface of Mis12^Mtw1^ are lethal in cells and disrupt interactions with CENP-U^Ame1^. This is further corroborated with N-terminal swap experiments suggesting no unique attribute of CENP- U^Ame1^ in binding the Mis12C^MIND^ and thus can be substituted with CENP-C^Mif2^ (Killinger et al., 2020).

It is noteworthy that the same Aurora B kinase-dependent control of Mis12C interaction with CENP-C and CENP-T has been shown to promote the recruitment and strengthen the binding of the Mis12C^MIND^ to CENP-U^Ame1^ (Figure 2C) (Dimitrova et al., 2016). Contrary to this, Hamilton and others (Hamilton et al., 2020) suggest that at least *in vitro* CENP-U^Ame1^ does not require the alleviation of Dsn1 autoinhibition for tight binding to Mis12C. Why the conflicting reports is not clear? Owing to the fact that mutation of residues S240 and S250 to alanine on Dsn1 renders cells harboring the URA-CEN plasmid inviable favors the model that both CENP-C^Mif2^ and the CENP-U^Ame1^ pathways fall under the influence of the Dsn1 autoinhibition-Aurora B regulatory system in budding yeast (Akiyoshi et al., 2013). Taken together, this suggests that the Aurora B phosphorylation-dependent removal of Dsn1 auto-inhibition is a critical step in outer kinetochore recruitment onto a linker platform and is conserved through Opisthokonta evolution (Figures 2A–C).

## 5 Kinetochore Architecture Based on Functional Linker Modules

Several linkages between the outer kinetochore and centromeric chromatin are reported as highlighted above (Figures 2A–C). Yet, it is unclear what evolutionary constraints drive the dominance/retention of specific pathways within each kinetochore ensemble thereby greatly influencing kinetochore architecture. In this section, we discuss some of the currently known strategies employed by organisms as well as ones that are likely, as alluded to through experimental and computational analysis. However, owing to the conservation of redundant pathways in certain systems, it is possible that the non-dominant but conserved pathways may be critical at an unresolved cellular context.

### 5.1 CENP-C-Pathway Dominant Kinetochores

**FIGURE 3:**
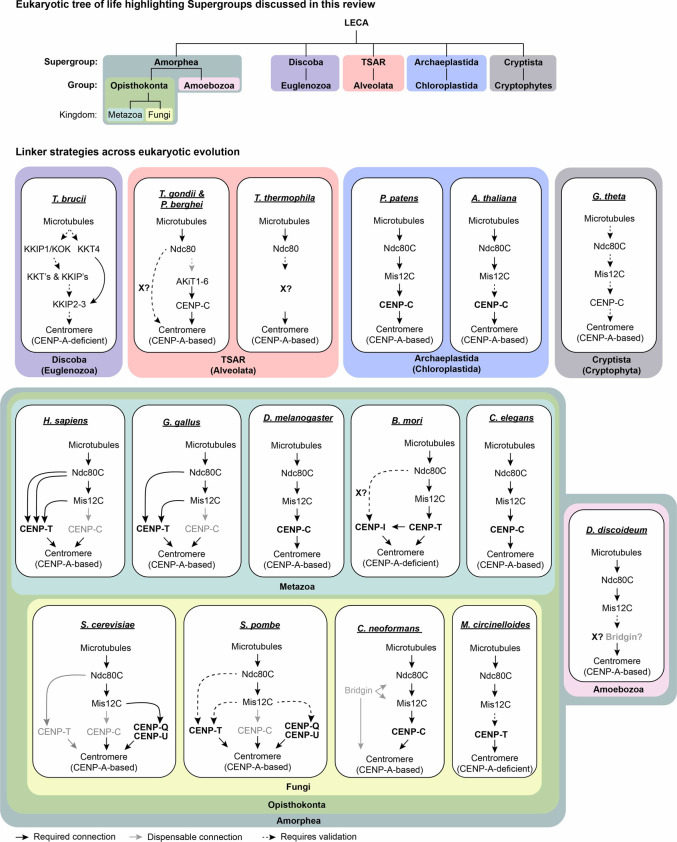
Linker strategies used across eukaryotic evolution to recruit the near-ubiquitous outer kinetochore. (Top) A cartoon highlighting the phylogenetic relationship between supergroups of representative species described in the bottom panel. (Bottom) The connection between the outer kinetochore centromeric chromatin across representative species is highlighted. The major linker pathway protein is highlighted in bold. Phylogenetic groups are mentioned in brackets where applicable. Names of kinetochore homologs are presented in the vertebrate format.

Kinetochores containing the CENP-C module were some of the earliest described systems. The functional module of the CENP-C linker pathway consists of CENP-A-CENP-C-Mis12C. Recent findings from the Apicomplexan kinetochore however report the loss of the Mis12C, while retaining the CENP-A-CENP-C axis (Brusini et al., 2021a), suggesting plasticity of this linker module. Amongst the described model systems consisting of this functional unit, it is only in the systems of *D. melanogaster* and *Caenorhabditis elegans* that CENP-C is the major pathway (Figure 3). It so happens that CENP-C is also the exclusive linker protein present in the two systems. A recent work on the basidiomycete *Cryptococcus neoformans* also highlights CENP-C as the sole CCAN component retained and likely the dominant pathway albeit with certain adaptations (Figures 2D, 3) (Sridhar et al., 2021).

#### 5.1.1 CENP-C Pathway Exclusive Systems

Extensive genetic and biochemical analysis of the *D. melanogaster* and *C. elegans* systems have failed to reveal other components of the CCAN but for CENP-C (Barth et al., 2014; Cheeseman et al., 2004; Goshima et al., 2007; Przewloka et al., 2007; Przewloka et al., 2011). The loss of CCAN components in a Dipteran ancestor is suggested to have taken place ∼250 million years ago (Drinnenberg and Akiyoshi, 2017). In the fruit fly, *Dm*CENP-C is essential (Heeger et al., 2005), and interactions with the Mis12C have been shown to be critical for accurate chromosome segregation (Przewloka et al., 2011). Notably in *D. melanogaster,* the Mis12C component Dsn1 was suspected to be lost (Przewloka et al., 2007; Liu et al., 2016). However, more recent bioinformatic predictions suggest the previously identified *Dm*Nsl1 as the *Dm*Dsn1 homolog. Interestingly, *Dm*Dsn1 lacks the N-terminal region including the basic autoinhibitory motif (Hooff et al., 2017; Plowman et al., 2019). Additionally, two functionally redundant Nnf1 paralogs exist. Further Knl1^Spc105^ was required for Mis12 complex assembly (Venkei et al., 2012). It is difficult to determine if these variations arose as a consequence of CCAN loss.

Ectopic targeting of the N-terminal domain of *Dm*CENP-C was shown to be sufficient to recruit the outer kinetochore. Using FRET and Talin-rod tension-sensing assays *Dm*CENP-C was shown to not only be sufficient for recruiting the KMN network but also in transmitting spindle forces (Ye et al., 2016).

Studies on the holocentric chromosome-containing nematode worm, *C. elegans*, also suggest a strong reliance on CENP-C^HCP−4^ for kinetochore assembly and accurate chromosome segregation, phenocopying CENP-A (Oegema et al., 2001; Desai et al., 2003). The absence of other CCAN components, a failure to identify novel components, and the importance of CENP-C^HCP−4^ in kinetochore assembly, together suggest CENP-C^HCP−4^ as a major linker pathway in *C. elegans* (Figure 3). Strikingly, during meiotic divisions both CENP-A and CENP-C^HCP−4^ have been reported to be dispensable for chromosome segregation (Monen et al., 2005). What retains the persistent outer kinetochore on these holocentric chromosomes is yet unknown.

Kinetochore composition in plants of the group Archaeplastida may resemble that of *D. melanogaster* or *C. elegans* where most CCAN components have not been identified (Hooff et al., 2017; Yamada and Goshima, 2017). However, experimental approaches to address plant kinetochore composition are limited and further analysis is required to conclude the absence of the CCAN in Archaeplastida. While CENP-S-X was identified in *P. patens*, kinetochore localization was not observed and no CENP-T-W homolog is reported (Figure 3) (Kozgunova et al., 2019). Similarly, while a CENP-O homolog is predicted, kinetochore localization of this protein was unclear (Kozgunova et al., 2019). CENP-C remains the only conserved linker component present. Knockdown of components of the CENP-C functional module phenocopied each other resulting in kinetochore malfunction in *P. patens*. Interestingly, while CENP-C has been described as a constitutive centromere localizing protein in Maize and *Arabidopsis thaliana* (Dawe et al., 1999; Ogura et al., 2004; Kozgunova et al., 2019), in the single-cell system of *P. patens*, loss of its localization from centromeres was observed soon after M-phase (Kozgunova et al., 2019).

#### 5.1.2 The *C. neoformans* Kinetochore

The kinetochore composition of the pathogenic basidiomycete *C. neoformans* echoes that of *D. melanogaster* or *C. elegans* where it is predicted to have lost all but CENP-C^Mif2^ of the CCAN, while retaining CENP-A^Cse4^ and a conserved outer kinetochore (Figure 2D) (Schleiffer et al., 2012; Hooff et al., 2017; Sridhar et al., 2017). CENP-C^Mif2^ is essential for viability, and a conditional knockout results in kinetochore collapse and increased segregation defects (Sridhar et al., 2021). To identify if additional kinetochore factors exist and to validate predictions, IP-MS studies of known kinetochore proteins CENP-C^Mif2^, Dsn1 and Spc25 were carried out. This identified all previously predicted kinetochore proteins, including CENP-C^Mif2^ but none of the other CCAN components. Interestingly, upon screening for other possible kinetochore proteins, an FHA-domain containing 1,295 aa long protein, named bridgin (Bgi1), was identified to specifically localize to the kinetochore from G2 to M-phase, peaking in anaphase (Figure 2D) (Sridhar et al., 2021). Sharing kinetochore localization dynamics similar to the KMN network it was observed that bridgin depended on the KMN network components for its localization. This required the FHA-domain in addition to a subsequent middle disordered region (Figure 2D). Strikingly, upon domain analysis, it was observed that the C-terminal basic region of bridgin could interact non-specifically with DNA and reconstituted nucleosomes. This was supported by the observation that chromatin-associated factors were enriched in IP-MS fractions of Bgi1 full-length over its mutant lacking the basic C-terminal region. Since *in vivo*, exclusive recruitment of bridgin to the kinetochore was observed, it was concluded that specific interactions with centromeric chromatin identified by native-ChIP were initiated following its recruitment by the outer kinetochore (Figure 2D) (Sridhar et al., 2021).

While bridgin is dispensable for growth, it is critical for accurate chromosome segregation, and its absence results in increased sensitivity to microtubule-depolymerizing factors. Co-evolution analysis suggested that the retention of bridgin across basidiomycetes is correlated with the loss of other potential linker pathways such as CENP-T^Cnn1^/-Q^Okp1^-U^Ame1^ (Sridhar et al., 2021). While CENP-C^Mif2^ is the dominant pathway in *C. neoformans*, we suspect a unique scenario where the outer kinetochore might have evolved to recruit a linkage, *via* bridgin, connecting it to centromeric chromatin (Figure 3). This may facilitate the reinforcement of the CENP-C^Mif2^ pathway critically at stages of high spindle tension such as anaphase when bridgin is most abundant at the kinetochore. Further insights into the transmission of force through this linkage would be interesting.

Curiously, bridgin is not only conserved across basidiomycetous fungi but also amongst other fungal phyla. Outside fungi, bridgin-like proteins are also identified in Ameobazoa (Figure 3). Amongst metazoans, the mitotic protein Ki-67 known to behave as a biological surfactant (Cuylen et al., 2016), shares bridgin-like features, thus suggesting functional divergence from a common ancestor (Sridhar et al., 2021).

### 5.2 CENP-T-Pathway Dominant Kinetochores

Soon after its characterization, tethering of CENP-T was shown to be sufficient to form a functional kinetochore at an ectopic locus (Hori et al., 2013). Yet, it is only recent studies that have shed light on the CENP-T linker pathway as being a viable mechanism at the native kinetochore to transmit force in not only systems that have lost the CENP-A-CENP-C module, as in the silk moth *B. mori* and Mucorales fungi *M. circinelloides* (Figure 3) (Navarro-Mendoza et al., 2019; Cortes-Silva et al., 2020), but also in well-studied vertebrate model systems such as the case with chicken DT40 cells (Figure 3) (Hara et al., 2018). We propose the dominance of the CENP-T linker pathway in the human and *Xenopus* systems as well (Figure 3) (see bellow). Thus, could the CENP-T dominant pathway be a feature of vertebrate kinetochores? CENP-T-Ndc80C module forms the most conserved core of this pathway.

#### 5.2.1 CENP-T Pathway Dominant Systems

The N-terminal regions of CENP-T and CENP-C independently were shown to be capable of forming a functional kinetochore at an ectopic locus in the chicken DT40 system. This generated artificial kinetochore enabled chromosome segregation in the absence of the native centromeric loci (Hori et al., 2013). Yet within the context of the native kinetochore, it was observed that the N-terminal Mis12C binding region of CENP-C was dispensable, and the growth of the mutant lacking the N-terminal region of CENP-C was comparable to wild-type cells. On the other hand, the Mis12C and Ndc80C binding domains of CENP-T were essential for cell viability. Further, using a tension-sensor system it was shown that CENP-T but not CENP-C could effectively transmit spindle pulling forces during M-phase (Hara et al., 2018). Further evaluating the roles of KMN network proteins on CENP-T, it was recently shown that two Ndc80C on CENP-T recruited either directly or as part of the KMN network were required for chromosome segregation (Takenoshita et al., 2022). Taken together, this suggests that in chicken DT40 cells CENP-T functions as the dominant pathway even in the presence of a functional CENP-C module. It is of interest to understand why the equivalent KMN recruitment on CENP-T and CENP-C may have variable essentialities.

Soon after the description of the CENP-T reliant chicken DT40 kinetochore, *in vivo* evidence for the retention of a single canonical pathway through CENP-T while losing CENP-C/-Q-U, along with CENP-A, in the *B. mori* and *M. circinelloides* was reported (Navarro-Mendoza et al., 2019; Cortes-Silva et al., 2020). Analysis of the remarkable system of *B. mori* by the Drinnenberg lab through IP-MS analysis of several kinetochore components identified the KMN network components except for Zwint-1. At the inner kinetochore, no homologs of CENP-A or CENP-C were detected. Homologs for CCAN components CENP-O-P and CENP-H were identified, yet with very limited homologies, thus requiring further verification. Additionally, while CENP-T, -S, and -X were identified, surprisingly no CENP-W homolog was observed, a key component influencing CENP-T localization in other systems. CENP-T itself was found to be essential for cell viability, requiring its N-terminal and histone fold domains to function. Further, CENP-T was found to be sufficient for recruiting the outer kinetochore complex. However, conditional depletion of CENP-T was insufficient to eliminate all the Ndc80C at the kinetochore, although the loss of Mis12C was almost absolute. A complete loss of the Ndc80C was observed only in CENP-I knockdown, a component of the *Bm*CENP-K-I-M complex on which CENP-T is also dependent (Figure 3) (Cortes-Silva et al., 2020). Thus, while CENP-T may account for an essential linker pathway in *B. mori*, the possible contribution of other pathways either directly or indirectly influenced by CENP-I cannot be ruled out. CENP-T has been identified in other related CENP-A-deficient insects that have also lost CENP-C (Cortes-Silva et al., 2020).

In the human pathogen, *M. circinelloides,* of the fungal subphylum Mucoromycotina, the CCAN components including CENP-T-W-S-X, along with CENP-H-I-K-M, CENP-L-N, and CENP-O-P have been identified, while CENP-A and CENP-C are absent. CENP-T was observed to be constitutively centromere localized (Figure 3). ChIP-seq analysis of the Mis12C components shows that kinetochores form on small mosaic centromeres, exhibiting features echoing that of budding yeast and fungal regional centromeres (Navarro-Mendoza et al., 2019). With the absence of CENP-C and CENP-Q-U proteins, CENP-T is the only other linker protein identified. Thus, addressing the contribution of the histone-fold containing CENP-T complex not only towards kinetochore function but also centromere establishment should yield some exciting results.

#### 5.2.2 Other Likely CENP-T Pathway Dominant Kinetochores:

##### 5.2.2.1 H. sapiens

In human cell lines, CENP-C and CENP-T are essential for cell viability and are independently capable of recruiting the outer kinetochore to an ectopic locus (Gascoigne et al., 2011; Rago et al., 2015; Musacchio and Desai, 2017). Over-expression of N-terminal outer kinetochore interacting motif of CENP-C in CENP-C-deficient HeLa cells disrupted outer kinetochore assembly (Screpanti et al., 2011). While it is possible that the CENP-C pathway plays a crucial role at the kinetochore, more recent evidence points to the kinetochore in human cells as being CENP-T biased. CENP-T has been shown to recruit twice as many Ndc80C at native kinetochores in comparison to the more abundant CENP-C (Suzuki et al., 2015). Additionally, CENP-T “stretching,” an indicator of propagating spindle forces is reported during M-phase (Uchida et al., 2021). While, a majority of the CENP-C N-terminal region was found to remain within CENP-A chromatin, likely unbound to the outer kinetochore (Suzuki et al., 2014). Further, alleviating Dsn1 autoinhibition was shown to be required for effective recruitment of the Mis12C to CENP-C (Kim and Yu, 2015; Rago et al., 2015). This scenario is comparable to *S. cerevisiae* and chicken where the N-terminal domain of CENP-C is dispensable. Additionally, in the human system, CENP-Q-U has been shown to be incapable of interacting with the Mis12C (Pesenti et al., 2018). Although CENP-C has a greater influence over CENP-T localization in comparison to chicken cells (Watanabe et al., 2019), it is likely a feature independent of its linker function. Considered together we speculate that it is probable that human kinetochore is biased towards the CENP-T linker pathway (Figure 3).

##### 5.2.2.2 Xenopus leavis

In egg extracts of the African clawed frog, *X. leavis,* CENP-C is capable of binding to CENP-A to mediate the recruitment of the Mis12C. Conserved is the feature where the outer kinetochore assembly on CENP-C was entirely dependent on phosphorylation of Dsn1 by Aurora B (Bonner et al., 2019). Although CENP-C partially influences CENP-T recruitment, both Ndc80C and Mis12C are localized at kinetochores, upon CENP-C depletion. Owing to the influence of the Dsn1 autoinhibitory motif on CENP-C recruitment and retention of outer kinetochore components upon CENP-C depletion, we favor a CENP-T biased kinetochore in *X. leavis* although *in vivo* experiments are required to conclude the same.

### 5.3 CENP-Q^Okp1^-U^Ame1^-Pathway Dominant Kinetochores

CENP-Q^Okp1^-U^Ame1^ dominant kinetochores have been exclusively described in the point-centromere containing budding yeast of *S. cerevisiae* and are likely to exist in closely related species. Although the entire suit of known linker components including CENP-T^Cnn1^ and CENP-C^Mif2^ has been described in budding yeast, CENP-T^Cnn1^-null cells are viable without strong chromosome segregation phenotype and loss of the CENP-C^Mif2^ N-terminus was shown to be well-tolerated only causing a minor growth defect in the presence of the microtubule poison benomyl (Cohen et al., 2008; Bock et al., 2012; Hornung et al., 2014). On the other hand deletions of either the Mis12C^MIND^ interacting motif on CENP-U^Ame1^ or the CENP-A^Cse4^ interacting domain on CENP-Q^Okp1^ were found to be essential for viability (Hornung et al., 2014; Fischböck-Halwachs et al., 2019). *In vitro* reconstitution studies reveals that CENP-Q^Okp1^U^Ame1^ can transmit mitotic relevant forces from the Mis12C^MIND^ to the centromeric nucleosome, making the CENP-A^Cse4^-CENP-Q^Okp1^-U^Ame1^-Mis12C^MIND^ the functional module of this pathway (Figure 3) (Hamilton et al., 2020).

It is suggested that CENP-Q-U originated through duplication of Mis12C components early in eukaryotic evolution. Although the CENP-Q-U is conserved in vertebrates as part of the larger CENP-O-P-Q-U-R complex, it does not function as a linker (Tromer et al., 2019). While CENP-U has been shown to be essential during mouse embryogenesis and in embryonic stem (ES) cells, it is found to be dispensable for mouse fibroblast cells and in chicken DT40 cells (Okada et al., 2006; Hori et al., 2008b; Kagawa et al., 2014). No interaction of this complex with the Mis12C has been reported (Hornung et al., 2014; Pesenti et al., 2018). Thus, the essentiality of CENP-U in development and ES cells might reflect functions other than a linker role. Indeed, vertebrate CENP-Q has been attributed to have microtubule-binding activities (Amaro et al., 2010; Pesenti et al., 2018), and has also been shown to recruit the mitotic kinesin CENP-E (Bancroft et al., 2015). Further, CENP-U primed by Cdk1 is one of the main Plk1 receptor sites at the kinetochore (Kang et al., 2011; Chen et al., 2021; Nguyen et al., 2021; Singh et al., 2021).

Thus, unlike other CCAN subcomplexes, CENP-Q-U seems to have undergone drastic functional remodeling at the kinetochore. This is further exhibited in its kinetochore interdependencies, where at the vertebrate kinetochore the CENP-Q-U complex is downstream of most CCAN components except for CENP-R, including the CENP-H-I-K and CENP-T-W-S-X complexes (Hori et al., 2008b), while in budding yeast all but CENP-C^Mif2^ depend on it (McKinley et al., 2015; Pekgöz Altunkaya et al., 2016). Taken together this makes CENP-Q^Okp1^-U^Ame1^ based kinetochores a unique feature of point-centromere based kinetochores.

### 5.4 Some Notable Kinetochores With Undefined Linker Pathways

#### 5.4.1 *S. pombe* (Supergroup: Amorphea, Group: Opisthokonta)

In the fission yeast model system, homologs for CENP-T^Cnp20^, CENP-C^Cnp3,^ and CENP-Q^Fta7^-U^Mis17^ have been identified, in addition to a full-complement of known inner and other kinetochore proteins similar to budding yeast. In a surprise turn of events, CENP-C^Cnp3^-null cells are viable, and observed chromosome segregation defects were largely suppressed by CENP-L^Fta1^ over-expression (Tanaka et al., 2009). This suggests that the primary role of CENP-C^Cnp3^ is in recruiting CENP-L^Fta1^ to the kinetochore, although it was recently reported to function as a receptor of the Mis12C (Zhou et al., 2017). CENP-T^Cnp20^ and CENP-Q^Fta7^-U^Mis17^ are essential for viability (Kim et al., 2010; Tanaka et al., 2009; Hayles et al., 2013). Yet surprisingly, temperature-sensitive mutants of either CENP-T^Cnp20^ or CENP-C^Cnp3^ did not significantly reduce the levels of the Ndc80C at the kinetochore (Tanaka et al., 2009). Further, IP-MS of CENP-U^Mis17^ failed to abundantly identify components of the KMN network (Shiroiwa et al., 2011). Interestingly, in *S. pombe,* a Nuf2 temperature-sensitive (*ts*) mutant has been shown to influence the localization of several inner kinetochore components including CENP-K^Sim4^ and CENP-I^Mis6^ (Saitoh et al., 2005), and Mis12 has been described to affect inner centromere structure. One possibility arising from these results may suggest that a redundant dependence upon linker proteins may exist between the equally abundant CENP-T^Cnp20^ and CENP-Q^Fta7^-U^Mis17^ at the fission yeast kinetochores (Figure 3) (Virant et al., 2021). Further analysis is required to shed light on the fission yeast kinetochore architecture.

#### 5.4.2 *Dictyostelium discoideum* (Supergroup Amorphea, Group: Amoebozoa)

Amongst the group Amoebozoa, *D. discoideum* has been reported to contain a conserved KMN network, CENP-A, and components of the CCAN including CENP-H, -I, -K, -M, -L, -N, -O, -P, -S, and -X. Linker components including CENP-C, -T-W, and -Q-U have not been identified (Hooff et al., 2017; Plowman et al., 2019). Interestingly, a homolog of the recently identified *C. neoformans* kinetochore component, bridgin, has been found (Figure 3) (Sridhar et al., 2021). The only characterized kinetochore protein, CENP-A^H3v1^ has been shown to be incorporated into centromeric chromatin (Dubin et al., 2010). Yet, unlike other characterized CENP-A, *Dd*CENP-A does not contain a longer loop1 region which is critical for centromere targeting in other systems (Black and Cleveland, 2011). However, it contains a long N-terminal unstructured region (Dubin et al., 2010), whose function has not been characterized.

#### 5.4.3 Apicomplexans [Supergroup TSAR (Telonemids, Stramenopiles, Alveolates, and Rhizaria), Group: Alveolata]

Apicomplexans along with ciliates and dinoflagellates belong to the Alveolata group. They contain parasitic species which include causative agents of toxoplasmosis and malaria (del Campo et al., 2019). These systems have been reported to harbor CENP-A and a conserved Ndc80C, but little else is known (Hooff et al., 2017; Zeeshan et al., 2020). Recent biochemical analysis however revealed the presence of two distinct CENP-C genes, in addition to the previously described SEA1, in *Plasmodium berghei* while *Toxoplasma gondii* contains one, they all being essential for cell proliferation (Bushell et al., 2017; Verma and Surolia, 2014; Zhang et al., 2018; Brusini et al., 2021a). Other CCAN components are likely absent. Interestingly, the Mis12C is likely also lost, while the SkaC is retained (Hooff et al., 2017; Brusini et al., 2021a). Cross-linked IP-MS of kinetochore components revealed the presence of AKiT1-6 sub-complex that likely positions itself between CENP-C and the outer kinetochore, resembling Mis12C and Knl1C positions at the kinetochore. Although AKiT1 depends on CENP-C for its kinetochore localization in *T. gondii*, neither protein depletion affected Nuf2 levels (Figure 3) (Brusini et al., 2021a). Thus, it is likely that other uncharacterized AKiTs or as yet undefined factors play a role to bridge the Ndc80C onto centromeric chromatin in addition to the redundant CENP-C pathway, highlighting the existence of a possible novel linker module (Figure 3).

#### 5.4.4 *Tetrahymena thermophila* (Supergroup TSAR, Group: Alveolata)

In the ciliate protozoan system of *T. thermophila*, only the centromeric determinant CENP-A and microtubule interacting Ndc80 are identified (Figure 3) (Cervantes et al., 2006; Cui and Gorovsky, 2006; Hooff et al., 2017). CENP-A^CNA1^ was shown to be localized as distinct spots to the peripheral centromeres in the micronucleus but absent in the macronucleus during vegetative growth. While in the meiotic prophase CENP-A^CNA1^ was observed to localize along the chromosome as puncta followed by a diffused phase as conjugation proceeds and subsequently reverting as distinct puncta. CENP-A^CNA1^ is described to be essential for accurate chromosome segregation (Cervantes et al., 2006).

#### 5.4.5 *Guillardia theta* (Supergroup Cryptista, Group: Cryptophytas)

The cryptophytes are a product of secondary endosymbiosis of a red algae and a eukaryotic host. Interestingly, like other cryptophytes, *G. theta* still harbors the nucleus (nucleomorph) and cytoplasm of their algal endosymbiont. This nucleomorph was shown to contain CENP-A, albeit lacking the extended loop1 region. The presence of a centromere and relict mitotic apparatus is suggested despite evidence lacking for the existence of a spindle (Douglas et al., 2001). The nucleomorph exists in a highly reduced and simplified form. Thus, their nuclear genomes are repositories for thousands of endosymbiont-derived genes. While rather little is known about the kinetochores of Cryptophytes or other members of the Cryptista supergroup, recent bioinformatic analysis of the nuclear genome has identified CENP-C, CENP-I, and CENP-K of the CCAN, most components of the outer kinetochore including components of the SkaC and Dam1C (Figure 3) (Hooff et al., 2017; Plowman et al., 2019). Interestingly, the Dam1C might have been derived from the secondary endosymbiont through horizontal gene transfer (Drinnenberg and Akiyoshi, 2017; Hooff et al., 2017; van Hooff et al., 2017).

#### 5.4.6 Kinetoplastids (Supergroup: Discoba, Group: Euglenozoa)

The kinetoplastids, which comprise a widespread sub-group of flagellated protozoans, surprised the kinetochore research field, with the identification of highly divergent kinetochores present in one of the earliest-branching eukaryotes (Akiyoshi, 2016). Unlike the other systems mentioned above, kinetochore structural components discovered in *Trypanosoma brucei* and other related kinetoplastids do not resemble any other reported proteins including CENP-A, but for KKIP1, a protein containing similarity to the coiled coils of Nuf2/Ndc80. Yet, chromosome segregation has been shown to rely on the conserved spindle microtubules (Akiyoshi and Gull, 2014; D’Archivio et al., 2017; Ersfeld and Gull, 1997). In contrast, they harbor a unique set of 36 kinetochore components, KKT1-20, KKT22-25, and KKIP1-12 (Akiyoshi and Gull, 2014; Nerusheva et al., 2019; Brusini et al., 2021b), where KKT16-18 were found to be similar to the axial element components of the synaptonemal complex. In addition the kinetochore shares microscopic similarity to the synaptonemal complex. These observations supports the hypothesis that kinetoplastid kinetochores repurposed parts of the meiotic machinery (Tromer et al., 2021).

Within the kinetochore, KKT2 and KKT3 contain a unique zinc-binding domain that is not only critical for their constitutive kinetochore localization but also exhibits weak DNA-binding (Akiyoshi and Gull, 2014; Marcianò et al., 2021). Recent studies further describe KKT4 as a multifunctional protein as it has been reported to be microtubule-binding with an additional DNA binding capability (Akiyoshi and Gull, 2014; Llauró et al., 2018; Ludzia et al., 2021). A recent work from the Wickstead lab shows that KKIP1 bridges the distinct inner and the outer kinetoplastid kinetochore (KOK, KKIP2-4,6,8–12) layers resulting in stretching while under metaphase tension (Figure 3) (Brusini et al., 2021b). Yet, speculating how the kinetochore is anchored in these kinetoplastids is a futile process and we await further insights into this system.

With these emerging results, we are excited and eagerly looking forward to research in this field towards unraveling the mysteries of these and other divergent kinetochores, including how the conserved outer kinetochore is linked to centromeric chromatin.

## 6 Discussion

In this review, we have highlighted the various strategies employed across eukaryotic evolution to ensure the recruitment and maintenance of the near ubiquitously conserved microtubule-interacting outer kinetochore (Figure 3). Tracing back the composition of the kinetochore in LECA suggests the likely presence of a full complement of known kinetochore proteins (Tromer et al., 2019). However, how was the LECA kinetochore organized? What was its actual composition and architecture? What and which linker pathways dominated? Since no eukaryote or proto-eukaryote that segregates in a LECA or pre-LECA manner has been discovered, addressing these questions is difficult. As simple prokaryotic systems only contain microtubule and centromere binding components, it is suggested that present-day kinetochore proteins, including the CCAN, were added later during eukaryogenesis. Shared ancestry with multiple other eukaryotic processes likely points to their origin sometime between the first eukaryotic common ancestor (FECA) and LECA (Tromer et al., 2019).

Factors such as the centromere drive, changes in spindle attachment dynamics, redundant linker pathways, and gene duplication events could have contributed to the kinetochore diversity observed today, originating from a full-compliment containing LECA kinetochore. Going from available phylogenies, it is needless to say that a lot of plasticity can be accommodated in this functionally conserved structure. The identification of new proto-eukaryotic, archeal or prokaryotic species might help in reconstructing kinetochore evolution.

The CENP-C functional pathway is likely the most conserved across evolution (Hooff et al., 2017; Plowman et al., 2019), yet surprisingly CENP-C-pathway dominant kinetochores reported to date are observed primarily in systems having lost other pathways. It remains a mystery as to what exactly the driving force is at kinetochores towards the maintenance of certain pathways, that in turn define their overall kinetochore architecture. Recent comparative analysis of the CENP-C^Mif2^ and CENP-Q^Okp1^-U^Ame1^ linker modules in budding yeast revealed both as being able to produce similar forces, although the CENP-Q^Okp1^-U^Ame1^ linkage more readily bound microtubules (Hamilton et al., 2020). Linker pathways act as an oligomerization platform for the Ndc80C, where copy numbers of the Ndc80C are greater than their linker counterparts (Joglekar et al., 2008; Suzuki et al., 2015; Dhatchinamoorthy et al., 2017; Cieslinski et al., 2021; Virant et al., 2021). Indeed, it is known that multivalency of the Ndc80C is required for effective tracking and force coupling (Volkov et al., 2018; Takenoshita et al., 2022), although the critical density sufficient for kinetochore functions is unknown. The ability to facilitate the Ndc80C oligomerization may be related to the dominance of one pathway over another. Despite the diverse and active studies in the kinetochore field, there exists limited data not only in the plethora of new but also in certain popular model systems, which needs to be bridged.

Interestingly, a common theme that seems to address some of the observed variations across linker pathways utilized is the role of Dsn1-mediated autoinhibition in regulating the interaction between CENP-C and the Mis12C (Figure 2A). The presence of multiple kinetochore linker pathways, such as direct recruitment of Ndc80C by CENP-T, is one strategy to overcome the reduction in CENP-C-Mis12C interaction (Kim and Yu, 2015; Hara et al., 2018; Lang et al., 2018). Alternatively, as observed in the single linker system of *D. melanogaster*, containing CENP-C exclusively, the autoinhibitory motif of *Dm*Dsn1 can be lost (Przewloka et al., 2009). While in *C. neoformans* an additional linkage *via* bridgin may aid in reinforcing the CENP-C pathway (Figure 2D) (Sridhar et al., 2021). Although no such strategy is reported in *C. elegans* we however believe that the Dsn1 inhibition is compensated for by a large number of attachments across its holocentric chromosomes. This is in line with CENP-C tethering assays across a large repetitive locus where CENP-C is shown to be sufficient to attract a functional outer kinetochore (Gascoigne et al., 2011; Hori et al., 2013; Rago et al., 2015), yet insufficient at the native kinetochore (Hara et al., 2018). Unfortunately, evidence is lacking as to why this regulation is conserved in Opisthokonts. Further validation and testing in real-time the influence of Dsn1 autoinhibition and Aurora B phosphorylation on the transmission of spindle forces across the CENP-C-Mis12C contact during the M-phase progression would be valuable. The requirement of this regulation to prevent undue connections at an ectopic loci is blurred by the recent discovery of intra-CENP-C^Mif2^ regulation. This preventing the recruitment of the outer kinetochore onto a CENP-C^Mif2^ platform in the absence of bound CENP-A^Cse4^ in *S. cerevisiae* (Killinger et al., 2020). Yet, given the studies across opisthokont model systems (Figure 3), the Dsn1-mediated autoinhibitory drive offers the best explanation for kinetochore architectures based on specific linker pathways. Conservation of the Aurora B phosphorylation-dependent CENP-C-Mis12C interaction is largely seen across eukaryotes, except for Excavates and Apicomplexans. Thus, this autoinhibitory-based regulation may well exist outside Opisthokonts (Plowman et al., 2019). It will be interesting to see if this hypothesis holds following future studies.

The presence of redundant including lineage-specific pathways, may allow for the loss of one or more of the linkages, as observed in basidiomycetous fungi (Sridhar et al., 2021). Once pathways known to exist in LECA are lost, the evolution of novel strategies is likely to arise as dictated by as yet unknown selective pressures during subsequent evolution (Akiyoshi, 2016; Cortes-Silva et al., 2020; Brusini et al., 2021a; Sridhar et al., 2021)*.* Alternatively, as mentioned in *D. melanogaster*, the kinetochore might balance compositional changes by altering the autoinhibitory regulation as observed with the *Dm*Dsn1 losing its auto-inhibitory motif.

Further insights into the functioning of well-studied pathways across systems may be obtained by evaluvating outlying phenotypes, such as how CENP-T performs its function in the absence of a CENP-W homolog in *B. mori* (Cortes-Silva et al., 2020), or the observation that CENP-T and -W might have variable temporal dynamics in *Xenopus* egg extracts (Krizaic et al., 2015). Additionally, evaluating how CENP-S-X loss phenocopies chromosome segregation defects of other kinetochore components without exhibiting centromere enrichment in the moss system *P. patens* (Kozgunova et al., 2019), may highlight additional adaptations of kinetochore sub-complexes.

Interestingly, the N-terminal region of CENP-C containing the outer kinetochore binding domain is highly conserved across various systems even where it is not the dominant linker pathway (Petrovic et al., 2016; Hara et al., 2018). Similarly, kinetochores often harbor multiple redundant pathways, although a dominant pathway is known. (Bock et al., 2012; Schleiffer et al., 2012; Sridhar et al., 2021). So why the apparent paradox? One possible explanation is that the conditions in which the “dispensable” pathways are essential have not been found. For example, the CENP-C pathway might be essential in specific developmental or differentiated stages in multicellular organisms, or under specific environmental growth conditions. These could include temperature or the presence of naturally occurring microtubule modulators in the unicellular environment which might affect spindle stability. Indeed, a large number of microtubule modulators have been identified from natural sources (Stanton et al., 2011). Supporting this possibility is the observation that the loss of redundant pathways even if they non-essential for viability, are important for accurate chromosome segregation under conditions of spindle stress (Bock et al., 2012; Schleiffer et al., 2012; Hornung et al., 2014; Sridhar et al., 2021). Thus screening of kinetochore determinants under more natural conditions may address these contradictions.

With the biochemical validation of a large number of kinetochore homologs, the variation particularly amongst CCAN components is evident. It is noteworthy that recent computational predictions have been rather robust in predicting the loss or retention of known kinetochore homologs, a testament to the improvements in computational methodologies but also that of genomics (Schleiffer et al., 2012; Hooff et al., 2017; Plowman et al., 2019; Tromer et al., 2019). However, going by recent studies, robust biochemical analysis of systems is key in identifying novel kinetochore candidates that cannot be identified computationally (Akiyoshi and Gull, 2014; Cortes-Silva et al., 2020; Brusini et al., 2021a; Sridhar et al., 2021).

While we have focused on the linker components of the CCAN in this study, several other kinetochore components also exhibit evolutionary lability and may play a role in influencing CCAN architecture or linker pathway function, such as CENP-R and its functional analog Nkp1-Nkp2 in budding yeasts, the small GTPase-like protein CENP-M (Basilico et al., 2014) which is essential in vertebrates yet lost in fungi, the role of outer kinetochore accessory proteins of the Dam1 and Ska complexes (Cheeseman, 2014), the point-centromere specific CBF3 complex critical for kinetochore nucleation (Biggins, 2013), or the mystical CENP-B, that binds to a specific DNA sequence in mammals (Hoffmann et al., 2016; Musacchio and Desai, 2017).

## 7 Future Perspective

With the elegantly posed “Ship of Theseus” analogy (Drinnenberg et al., 2016) to describe kinetochore plasticity, going forward it would be exciting to see the compositional diversity this structure can attain across disparate systems. These include not only the kinetochores of Metamonad Carpedirmonas, Diplonemids and Euglenids likely lacking most known kinetochore components, but also the divergent Apicomplexan outer kinetochore and in CENP-A deficient systems of *B. mori* and *M. circinelloides* (Butenko et al., 2020; Brusini et al., 2021a; Salas-Leiva et al., 2021; Tromer et al., 2021). In this review, we have highlighted systems from five of the thirteen newly defined eukaryotic supergroups (Burki et al., 2020). However, it should possible to target unique and divergent systems given the availability of rather robust computational prediction tools. In spite of the likely challenges of biological dissection in certain systems, it is an area that may yield fruitful and exciting results. With the abundance of information on the various components in model systems, it would be rewarding to look into conditions of their requirement and plasticity in varying circumstances mimicking the native environment and life cycle.

In addition to the likely identification of novel linker candidates, it is equally likely that known factors may be repurposed. A prime candidate for this would be CENP-I, which has been shown to not only localize closely with the Ndc80C in humans (Suzuki et al., 2014) and directly interact with it in budding yeast, chicken DT40 and human systems (Mikami et al., 2005; Kim and Yu, 2015; Pekgöz Altunkaya et al., 2016), but also to be capable of interacting non-specifically with DNA or reconstituted nucleosomes as part of the CENP-H-I-K-M complex (Weir et al., 2016; Pesenti et al., 2022). Additionally, CENP-I and its fission yeast homolog Mis6 are shown to be required for new CENP-A deposition (Takahashi et al., 2000; Okada et al., 2006). CENP-I has also been described to be able to nucleate the formation of a functional kinetochore when tethered at an ectopic locus (Hori et al., 2013), and more recently the CENP-I homolog in *B. mori* has been shown to be critical in kinetochore maintenance and recruiting the outer kinetochore (Cortes-Silva et al., 2020).

Thus, the pursuit of understanding how kinetochore plasticity is accommodated towards performing a conserved function is an exciting direction the kinetochore field is working towards.
